# Three-dimensional CT for the diagnosis and management of bipartite scaphoids: a report of four cases in three patients

**DOI:** 10.1177/17531934211053479

**Published:** 2021-10-20

**Authors:** Justine Dufour, Thierry Christen, Fabio Becce, Sébastien Durand

**Affiliations:** 1 Department of Hand Surgery, Lausanne University Hospital, Lausanne, Switzerland; 2 Department of Diagnostic and Interventional Radiology, Lausanne University Hospital and University of Lausanne, Lausanne, Switzerland

**Keywords:** Bipartite scaphoid, os centrale, anatomical variation, scaphoid nonunion, 3-D imaging, computed tomography

## Abstract

We investigated the role of three-dimensional (3-D) CT in the diagnosis and management of four bipartite scaphoids in three patients. We computed the volume ratio, moment of inertia ratio and direction vector from the centroid of the scaphoid to the os centrale carpi. We found that the os centrale carpi was always smaller than the scaphoid and showed an elongated shape in the scaphoid longitudinal axis. Its position was always posterior compared with the scaphoid anteroposterior axis. The main morphological feature of bipartite scaphoids was the continuity of the scaphoid from its proximal to distal aspect along the longitudinal axis. These criteria from 3-D imaging should be considered useful in the diagnosis of bipartite scaphoid as it allows differentiation from nonunion. 3-D single-photon emission computed tomography (SPECT)/CT was helpful in the surgical decision-making when the patient was symptomatic. 3-D imaging was also used for the preoperative simulation and planning of bone fusion as it simplifies surgery and makes it more accurate. Here we provide clear criteria for diagnosing bipartite scaphoids and for the planning when surgery is deemed necessary.

## Introduction

The scaphoid bone is formed by the fusion of the os centrale carpi and the radial chondrification centre in embryos measuring between 28 to 30 mm long (Gray, 1957). A bipartite scaphoid can be defined by the failure of fusion of these ossification centres; therefore, by definition, it is a congenital disorder. It is rarely seen in adults as its frequency in dissection ranges from 0.1% to 0.6% (Gruber, 1877; Pfitzner, 1900). However, Wolff denied the existence of this condition by reviewing all the documented cases presented by Gruber and Pfitzner (Wolff, 1903). He concluded that they were all scaphoid nonunions. The challenge in differentiating a bipartite scaphoid (i.e. scaphoid with a separate centrale component) from a scaphoid nonunion limits the ability of anatomists to properly evaluate the frequency and evolution of this finding and of clinicians to diagnose nonunions. Differentiating the remaining os centrale carpi from scaphoid nonunion has been a point of contention for almost a century (Louis et al., 1976). Several authors (Bunnell and Boyes, 1970; Jerre, 1947; Talesnik, 1985) have suggested various criteria for the diagnosis of bipartite scaphoid, such as bilateral partition, absence of history or sign of injury, clear space between the bony components with smooth edges at the joint surface, equal size and bone densification of each part and absence of degenerative changes in the radioscaphoid joint. Unfortunately, these criteria are not always sufficient to avoid misdiagnosing congenital bipartition and nonunion. The purpose of this study was to evaluate the role of 3-D imaging in the diagnosis and management of bipartite scaphoids.

## Methods

### Patients

**Figure 1.**
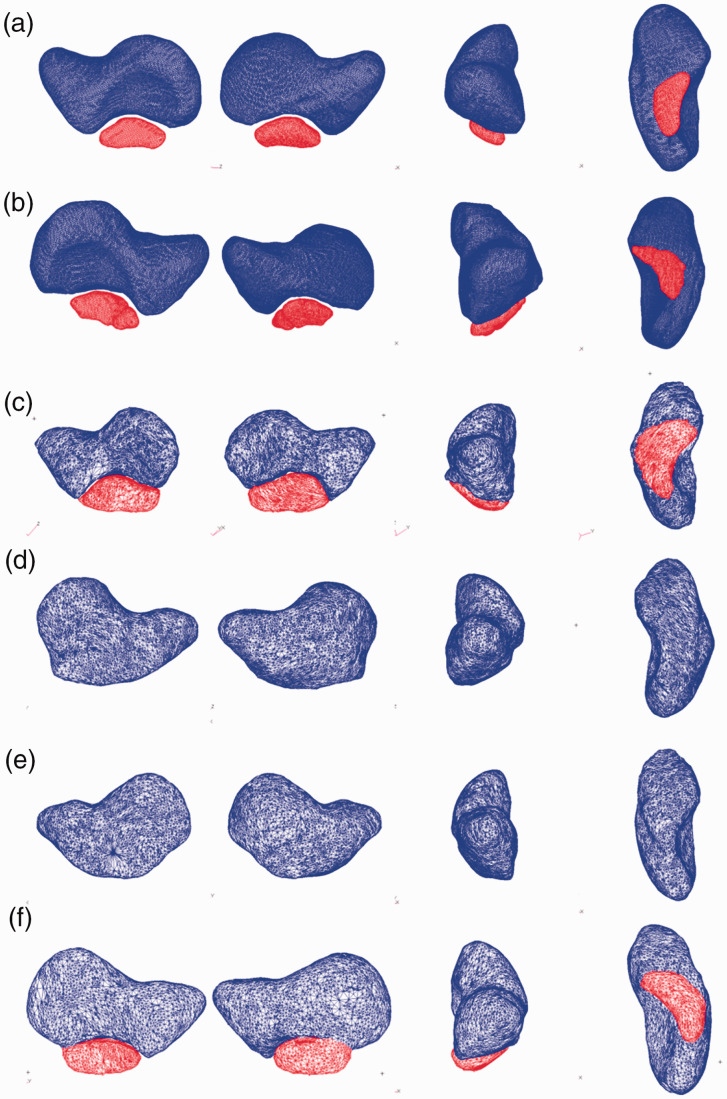
Right and left scaphoids in Patient 1 (a, b), Patient 2 (c, d) and Patient 3 (e, f). All scaphoids are shown in medial view (left), lateral view (second column), anterior view (third column) and inferior view (right). Blue ossicle: scaphoid; red ossicle: os centrale carpi.

Four bipartite scaphoids in three men with an average age of 34 years (Table 1) were incidentally observed between 2015 and 2021 on conventional radiographs of patients complaining of post-traumatic pain on the radial side of the wrist. One patient presented with bilateral scaphoid bipartition. The two others had a unilateral bipartite scaphoid with a ‘full scaphoid’ shape (Morsy et al., 2019) on the contralateral side (Figure 1).

**Table 1.** Patient demographics and clinical data.

	Patient 1	Patient 2	Patient 3
Bipartite scaphoid location	Both right and left wrists	Right wrist (full shape on the left wrist)	Left wrist (full shape on the right wrist)
Wrist pain Injury mechanism	Left wrist Twisting of the wrist	Right wrist Crush injury to the wrist	Left wrist Snowboard fall on the outstretched hand
Initial treatment	Casting 4 weeks Physiotherapy Steroid injection	Casting 4 weeks Physiotherapy Steroid injection	Casting 4 weeks Physiotherapy
SPECT/CT	Focal increased radiotracer uptake	Focal increased radiotracer uptake	—
Duration before surgery (months)	9	7	—
Surgery	Os centrale carpi excision	Os centrale carpi and scaphoid fusion with two 1.2-mm screws	—
Follow-up (months)	15	28	22
Grip strength (kg/cm^2^)	At follow-up R 44, L 35 Before surgery R 34, L12	At follow-up R25, L 34 Before surgery R 6, L 32	At follow-up R 60, L 46
Wrist flexion/extension (degrees)	At follow-up R 80-0-70, L 80-0-70 Before surgery R 65-0-65, L 50-0-55	At follow-up R 50-0-70, L 70-0-80 Before surgery R 50-0-40, L 70-0-80	At follow-up R 65-0-80, L 75-0-80
Return to work or activities and pain	100% returned as a scaffolding displayer, no pain	100% returned as a bricklayer, but pain during heavy work	Returned as a seller and javelin thrower, no pain

R: right; L: left; SPECT/CT: single-photon emission computed tomography.

### Imaging and 3-D image reconstruction

All wrists were scanned using multidetector CT systems with routine clinical protocols. Thin sections were reconstructed with voxels of ∼0.25 × 0.25 ×0.30 mm^3^ after interpolation. Wrist bone segmentation was performed using 3-D Slicer software (https://www.slicer.org). Briefly, the thresholding algorithm was used to produce an initial mask by separating bones from the surrounding soft tissues. The completed masks of each individual bone were used to create 3-D polygon models exported in stereolithography (STL) file format. The scaphoid and os centrale carpi polygonal models were exported (Figure 1), and 3-D quantification of the bipartite scaphoid (Table S1) was obtained using MSC.Patran 2005 r2 software (Newport Beach, CA, USA).

### 3-D quantification

**Figure 2.**
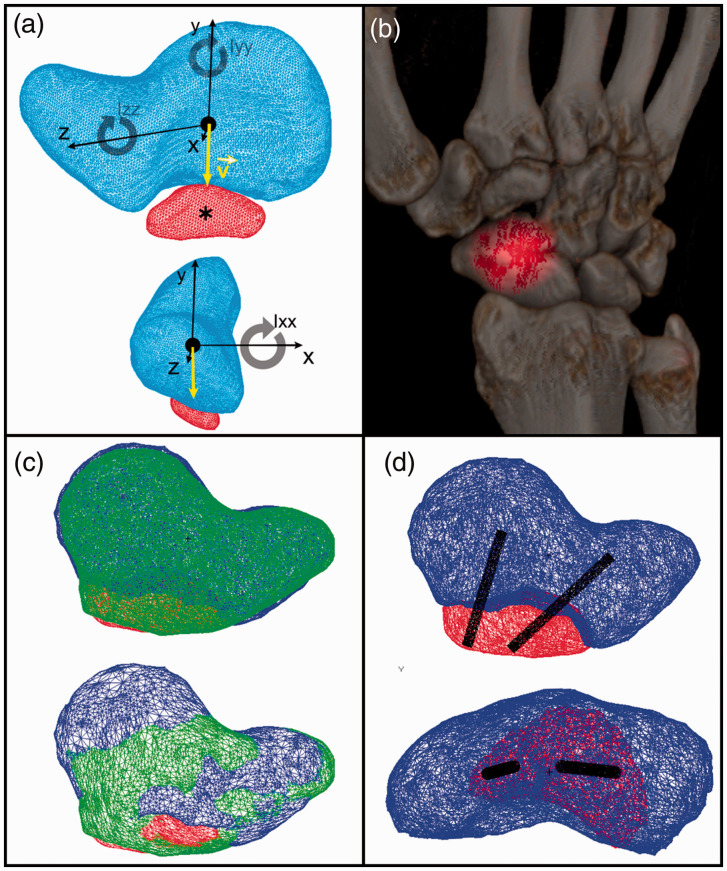
Centroids of the scaphoid (black round dot), os centrale carpi (asterisk), moments of inertia I_xx_, I_yy_, I_zz_ of the scaphoid along its three axes *x*, *y*, *z* are represented. The direction vector $\overset{\to }{v}$ (yellow) is drawn from the scaphoid centroid toward the os centrale carpi centroid (a). Focal increased radiotracer uptake in the os centrale carpi in Patient 2 using 3-D SPECT/CT scan with 99 m Tc-DPD (b). Comparison of the virtual 3-D model of the bipartite scaphoid (Patient 2) superimposed with the mirror model of the full shape contralateral scaphoid (green) (c). Simulation of the surgery using 1.2 mm cylinders (black lines). Orientation and size of the screws are estimated (d).

The volumes of the scaphoid, the os centrale carpi, the total volume and the os centrale carpi/scaphoid volume ratio were computed for each scaphoid. To quantify the geometry and indirect mass distribution, the symbols I_xx_, I_yy_, I_zz_ were used to express the moments of inertia of the scaphoid and os centrale carpi around their three axes (Figure 2(a)). The scaphoid centroid of each model was positioned at the origin (0, 0, 0) with the three principal axes corresponding to the Cartesian coordinate system. The position of the os centrale carpi relative to the scaphoid was calculated using the direction vector $\overset{\to }{v}$ . This vector (unit vector) at the origin of the coordinate system (scaphoid centroid) points toward the centroid of the os centrale carpi (Figure 2(a)).

All three patients were initially treated with conservative measures (Table 1). A single-photon emission computed tomography (SPECT)/CT scan with 99 m Tc-DPD was performed if pain persisted for 6 months. 3-D surgical simulation and planning was performed depending on the size of the os centrale carpi.

## Results

### Patient 1

**Figure 3.**
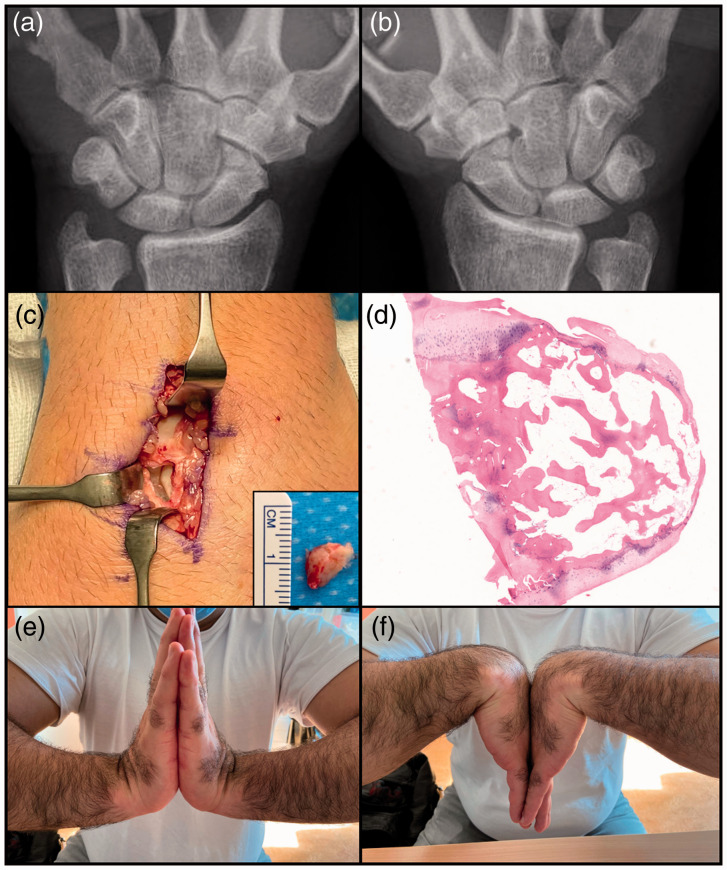
Patient 1. Posteroanterior radiographs of the left (a) and right wrists (b). Intraoperative photographs of the posterior aspect of the wrist shows the os centrale carpi after excision and the cartilaginous surface of the scaphoid adjacent to the os centrale carpi. (c) Histology confirmed the presence of hyaline cartilage around the entire os centrale carpi (d). Clinical results 6 months after surgery (e, f).

A 47-year-old, right-handed man presented with pain in his left wrist after a sprain. Posteroanterior radiographs (Figure 3(a), 3(b)) and 3-D reconstruction from CT (Figure 1(a) and (b)) demonstrated the presence of bilateral bipartite scaphoids. The os centrale carpi was smaller than the scaphoid on both sides. The moment of inertia ratio showed an elongated shape for both the scaphoid and os centrale carpi because of a higher distribution of mass around the *x*-axis and *y*-axis compared with the *z*-axis. The direction vector $\overset{\to }{v}$ was almost perpendicular to $\overset{\to }{z}$ and opposed to $\overset{\to }{y}$ . We found a continuity of the scaphoid from its proximal pole to the tubercule. Despite 9 months of conservative treatment (casting for 4 weeks, physiotherapy, steroid injection), the patient still experienced pain. A SPECT/CT scan with 99 m Tc-DPD showed focal increased radiotracer uptake. The os centrale carpi was excised (Figure 3(c)) due to its osteonecrotic appearance and its small volume compared with the scaphoid (14%). Presence of hyaline cartilage around the entire os centrale carpi was confirmed (Figure 3(d)).

### Patient 2

**Figure 4.**
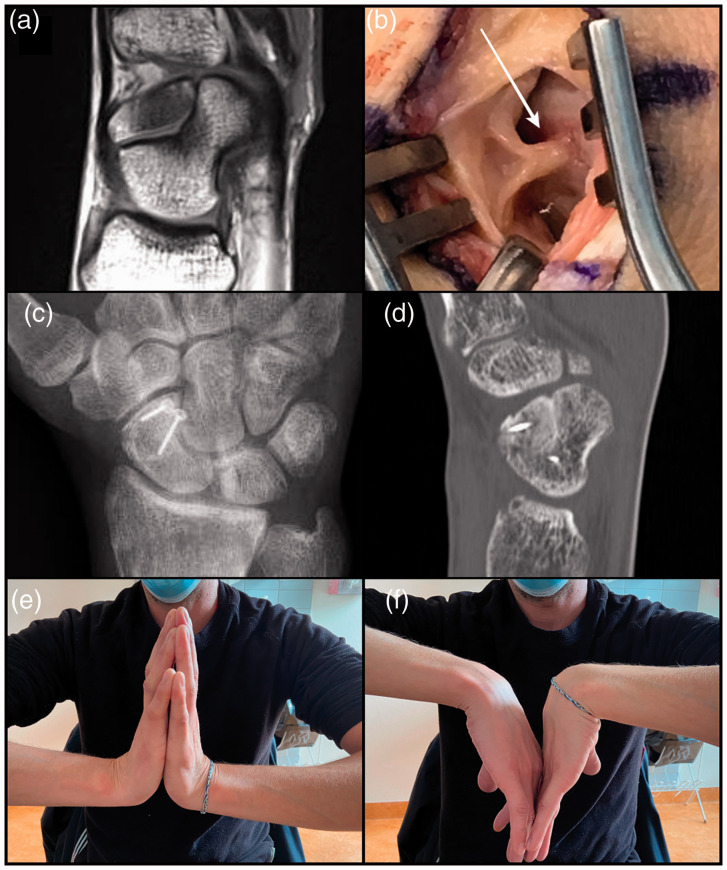
Patient 2. MRI showing the bipartite scaphoid, sagittal T1-weighted Turbo spin echo (TSE) sequence (a). Intraoperative photographs of the posterior aspect of the wrist showing a synovial fold (white arrow) entering the os centrale carpi/scaphoid joint (b). Posteroanterior radiographs of ossicles fusion using two 1.2-mm screws with washers (c). Confirmation of ossicles fusion on CT scan (d). Clinical results 18 months after surgery (e, f).

A 37-year-old, right-handed man presented with pain after a crush injury to his left wrist. MRI (Figure 4(a)) and 3-D reconstruction from CT (Figure 1(c)) demonstrated the presence of a right bipartite scaphoid. The left scaphoid (Figure 1(d)) displayed a full type shape according to a recent classification (Morsy et al., 2019). On the right side, the os centrale carpi was smaller than the scaphoid. The moment of inertia ratio showed an elongated shape for both the scaphoid and os centrale carpi, and the direction vector $\overset{\to }{v}$ was almost perpendicular to $\overset{\to }{z}$ and opposed to $\overset{\to }{y}$ . We found a continuity of the scaphoid from its proximal pole to the tubercule. Despite 7 months of conservative treatment (casting, physiotherapy, steroid injection), the patient still experienced pain. A SPECT/CT scan showed increased radiotracer uptake predominantly in the distal scaphoid region (Figure 2(b)). In this case, an os centrale carpi/scaphoid fusion was attempted (Figure 4(c) and (d)) since the volume of the os centrale carpi was relatively large (29%). 3-D surgical planning (Figure 2(c), 2(d)) allowed determination of the orientation, size and type of device to be used. A synovial fold entering the os centrale carpi/scaphoid joint was observed (Figure 4(b)) along with hyaline cartilage between the two ossicles. Fusion of the two ossicles was obtained after 4 months (Figure 4(c) and (d)).

### Patient 3

A 19-year-old, right-handed man presented with pain in the left wrist after a snowboard fall on the outstretched hand. 3-D reconstruction from the CT scan (Figure 1(e)) demonstrated a bipartite left scaphoid. The right scaphoid (Figure 1(f)) displayed a full type shape. On the left side, observations were similar to Patients 1 and 2 concerning the volume ratio, moment of inertia ratio, direction vector $\overset{\to }{v}$ and continuity of the scaphoid from its proximal pole to the tubercule. After 4 weeks of conservative treatment, the patient was free of pain. Results and detailed data are shown in Tables 1 and S1.

## Discussion

Previous publications have reported cases of bipartite scaphoid similar to ours (Abascal et al., 2001; Adolfsson, 2000; Hong et al., 2014; Yang et al., 1994). Others probably misinterpreted nonunion as bipartite scaphoid when their conventional radiographs are critically reviewed (Chang et al., 2013; Dubrana et al., 1999; Et-tai et al., 2008; Richard et al., 1987; Sherwin et al., 1971; Takemitsu et al., 2014). The accurate identification of bipartite scaphoid is important for management since its treatment differs from that of scaphoid fracture or nonunion (Sherbok and Grogan, 1980). We found that 3-D imaging with CT and/or SPECT/CT was an important and useful tool in the diagnosis of bipartite scaphoid.

This case series shows the existence of clear geometric/morphometric features shared by the os centrale carpi in four bipartite scaphoids from our three patients. The os centrale carpi was clearly smaller than the scaphoid. Its shape was elongated and its *z*-axis parallel to that of the scaphoid. The position of the os centrale carpi was always distal to the scaphoid according to the *y*-axis. The remaining os centrale carpi was located in the carpus between the capitate, the scaphoid and the trapezoid. The main morphological feature of bipartite scaphoids was the continuity of the bone from the proximal to the distal aspect along the *z*-axis, a feature not encountered in case of fracture or nonunion. If the os centrale carpi is removed, the scaphoid morphology appears normal and whole. This is not the case in nonunion of transverse, oblique and coronal fractures (Slutsky et al., 2016). If one fragment is taken out, the scaphoid appears incomplete and too short/small.

Variations of scaphoid shape have been studied by manual measurements between landmarks on dried bones (Ceri et al., 2004; Compson et al., 1994), 3-D models (Morsy et al., 2019) and statistically shaped models (Van de Giessen et al., 2010). Among various measurements, the height of the scaphoid waist shows the greatest variation, with 29% in a statistical shape model (Van de Giessen et al., 2010). This variability between full and slender scaphoids is present in many classifications (Morsy et al., 2019). In patients with unilateral bipartite scaphoid, we observed a full scaphoid shape of the contralateral side (Figure 1). We suppose that the os centrale carpi fusion during embryogenesis plays an important role in the scaphoid shape determination. If fusion does not occur or is delayed, it can affect the size and morphology of the scaphoid due to heterochrony (Kivell and Begun, 2007).

Additional ossicles may be found in the foot or the hand and can become painful, usually after trauma (Sacks, 1949). Since most of them are asymptomatic, it may be difficult to determine if they are responsible for the patient’s complaints. SPECT/CT imaging has given a new dimension to the routine planar bone scintigraphy. The ability to superimpose the detailed 3-D anatomy of a CT scan to the bone turnover/molecular data obtained by scintigraphy has provided insight into a variety of clinical conditions (Scharf, 2015; Tabotta et al. 2019). When accessory ossicles are associated with pain, focally increased radiotracer uptake at a symptomatic joint can corroborate the suspected diagnosis. Conversely, the absence of radiotracer uptake can reliably be interpreted as evidence that the anatomical anomaly is not likely the source of the pain. The 3-D SPECT/CT scans performed on Patients 1 and 2 were helpful in identifying focally increased radiotracer uptake in or near the os centrale carpi (Figure 2(b)). We believe this imaging technique may play a prognostic role in the surgical outcome.

Some authors suggest removing a symptomatic os centrale carpi (Abascal et al., 2001; Adolfsson, 2000; Hong et al., 2014) especially if it is fragmented or small. The issue seems different for patients with a large os centrale carpi that articulates in a major manner with the capitate and which has a full scaphoid shape on the contralateral side (Patient 2). In this case, the os centrale carpi could be excised, but we consider that an os centrale carpi/scaphoid fusion should be attempted from a biomechanical point of view.

3-D preoperative planning can simplify surgery and improve the procedure’s accuracy in case of scaphoid nonunion with marked deformity (Nagy, 2020). Comparison of the virtual 3-D models of the bipartite and healthy contralateral scaphoid (Figure 2(c)) facilitates the planning of the fusion and helps to determine the size and direction of the screws (Figure 2(d)). In our cases, we found that 3-D imaging provided a threefold interest in the presence of bipartite scaphoid. First and most importantly, it allows obtaining an accurate diagnosis since it can differentiate congenital bipartition from nonunion morphologically and morphometrically. Then, from a prognostic point of view, SPECT/CT may play a role by assessing the association between increased bone turnover/metabolism around an os centrale carpi and wrist pain. Finally, the 3-D modelling facilitates the surgical planning.

## Supplementary Material

Supplementary material
