# Supplementary material for: Three-dimensional CT for the diagnosis and management of bipartite scaphoids: a report of four cases in three patients
Source: J Hand Surg Eur Vol. 2021 Oct 20;47(3):264–9. doi: 10.1177/17531934211053479 (PMC8892043; doi:10.1177/17531934211053479)
Supplement: Supplementary material [file sj-pdf-1-jhs-10.1177_17531934211053479.pdf]

	Patient 1	Patient 2	Patient 3
Bipartite scaphoid location	Both sides	Right side (full shape on the left side)	Left side (full shape on the right side)
Number of elements (shell meshing)	41408 (Right)<br>54660 (Left)	24992 (Right)<br>17978 (Left)	13232 (Right)<br>13450 (Left)
Volume (cm^3)	Right (Sc: 1.13, C: 0.15, T:1.28)<br>Left (Sc: 1.13, C: 0.16, T: 1.29)	Right (Sc: 1.57, C: 0.46, T: 2.03)<br>Left (T: 1.63)	Left (Sc: 1.38, C: 0.26, T: 1.64)<br>Right (T: 1.57)
Os centrale carpi/scaphoid volume ratio (%)	Right (13.3)<br>Left (14.2)	Right (29.3)	Left (18.9)
**Ixx/Izz**	Right (Sc: 2.41, C: 2.74, T: 2.13)<br>Left (Sc: 2.48, C: 2.96, T: 2.14)	Right (Sc: 2.44, C: 2.27, T: 1.97)<br>Left (T: 2.21)	Left (Sc: 2.73, C: 2.81, T: 2.22)<br>Right (T: 2.33)
**Iyy/Izz**	Right (Sc: 2.24, C: 2.43, T: 1.80)<br>Left (Sc: 2.29, C: 2.53, T: 1.84)	Right (Sc: 2.32, C: 1.83, T: 1.64)<br>Left (T: 1.92)	Left (Sc: 2.56, C: 2.55, T: 1.84)<br>Right (T: 1.99)
**Ixx/Iyy**	Right (Sc: 1.08, C: 1.12, T: 1.18)<br>Left (Sc: 1.08, C: 1.17, T: 1.17)	Right (Sc: 1.05, C: 1.24, T: 1.21)<br>Left (T: 1.15)	Left (Sc: 1.07, C: 1.10, T: 1.21)<br>Right (T: 1.17)
**Direction** $\vec{v}(x, y, z)$	Right (0.16, -0.98, -0.15)<br>Left (0.31, -0.95, 0.03)	Right (0.09, -0.99, 0.01)	Left (-0.07, -0.99, 0.07)

Table S1: 3D quantification and morphometric data. Sc: scaphoid, C: os centrale carpi, T: total
